# Mad or mad-mad: conveying subtle emotion with face emoji

**DOI:** 10.3389/fpsyg.2023.1183299

**Published:** 2023-09-28

**Authors:** Sri Siddhi N. Upadhyay, Danielle N. Gunraj, Nicklas C. Phillips

**Affiliations:** ^1^Department of Psychology, James Madison University, Harrisonburg, VA, United States; ^2^Independent Researcher, Windsor, CT, United States

**Keywords:** emoji, texting, text comprehension, computer-mediated communication, pragmatics

## Abstract

**Introduction:** To compensate for the lack of pragmatic information available when communicating via text message, texters make frequent use of texting-specific cues, or *textisms*, to convey meaning that would otherwise be apparent in spoken conversation. Here, we explore how one such cue, face emoji, can impact the interpretation of text messages.

Methods: In Experiment 1, we paired neutral text messages with valenced face emoji to determine whether the emoji can alter the meaning of the text. In Experiment 2, we paired valenced text messages with valenced face emoji to determine whether the emoji can modulate the valence of the text.

Results: In Experiment 1, we found that texts paired with positive emoji were rated more positively than texts paired with negative emoji. Furthermore, texts paired with stronger-valenced emoji were rated as less neutral compared to texts paired with milder-valenced emoji. In Experiment 2, we found that slightly positive texts paired with strong positive emoji were rated somewhat similarly to the same texts paired with mild positive emoji; however, slightly negative texts paired with strong negative emoji were rated much more negatively than the same texts paired with mild negative emoji.

Discussion: These results indicate that the presence of face emoji, particularly negative face emoji, can alter the interpretation of text messages, allowing texters to communicate nuanced meaning and subtle emotion.

## Introduction

Modern life relies heavily on computer-mediated communication (CMC), such as email, social media chat, and text messaging. This reliance increased due to the COVID-19 pandemic, which forced much of the world to move work, school, and commerce largely online. CMC became a vital part of daily life as individuals attempted to combat social isolation while society tried to tackle a public health crisis. With this greater dependence on digital communication, the frequency of text messaging increased by 43% to become the prevalent, preferred, and even, primary form of communication when compared to other forms of digital communication, including email, voice calls, video calls, and social media (Nguyen et al., 2020).

Contrary to early research, which suggested that CMC is impoverished compared to face-to-face communication (Daft and Lengel, 1986), text messaging can effectively convey social and interpersonal information to allow for rich communication in a digital environment (McCormick and McCormick, 1992; Riordan and Kreuz, 2010; Kalman and Gergle, 2014). Text messaging, a form of written language, can stand in for face-to-face conversation because this type of digitalk (Turner, 2010) or talk-writing (McWhorter, 2012) mimics speech (Houghton et al., 2018). It is dynamic, fast-paced, and reciprocal, much in the way that spoken conversation facilitates turn-taking and the rapid exchanges of ideas and information (Darics, 2013).

The ubiquity of text messaging, or texting, has led to the evolution of linguistic cues specific to the texting environment. These cues are broadly referred to as *textisms* and they help texters create and interpret written messages more effectively and efficiently. They are especially important when the literal interpretation of the written message is not intended or is ambiguous–for example, when trying to convey humor (Hsieh and Tseng, 2017) or sarcasm (Filik et al., 2016; Thompson and Filik, 2016). Textisms convey pragmatic and social meaning, functioning in place of the nonverbal cues one might give and receive in face-to-face conversation, such as facial expressions, gestures, and tone of voice. Common types of textisms include existing linguistic cues that have been adapted to signal something new and more speech-like, such as punctuation (Gunraj et al., 2016; Houghton et al., 2018) and letter repetition (e.g., “okayyy” to mimic phoneme extension), as well as cues specific to texting and the needs of a digital communication environment, such as emoticons and, more recently, emoji (Kalman and Gergle, 2014).

Much of the existing work on textisms consists of corpus analyses of text messages that identify and classify naturally occurring textisms (e.g., Riordan and Kreuz, 2010; Baron and Ling, 2011; Darics, 2013; Kalman and Gergle, 2014). Nevertheless, more recent work has focused on experimental exploration. For example, both Gunraj et al. (2016) and Houghton et al. (2018) investigated the subtle meaning conveyed by punctuative periods. Their findings indicate that experimental studies can help illuminate the variety of cues used in text messaging.

A more recent focus for texting research involves the comprehension of emoticons and their more modern replacement, emoji. Unlike the period, these cues were created specifically for a CMC environment and may play several roles. For example, emoji may serve both a semantic and syntactic function (Heck, 2007; Hand et al., 2022). Previous research has demonstrated that the presence and the quantity of emoticons used within a message can impact the amount of attention the message receives; the more emoticons used, the greater the attention given to that message (Willoughby and Liu, 2018).

In addition to emoji quantity, emoji placement may also impact attention. Support for this claim is provided by Robus et al. (2020). Examining the impact of emoji placement on attention, they found that placing emoji at the beginning of a sentence resulted in more fixations, but placing emoji at the end of a sentence resulted in longer fixations (Robus et al., 2020). Placing emoji in a sentence-initial position is not common in everyday texting; thus, the novelty of their position may have increased the number of fixations. Furthermore, readers may have refixated the sentence-initial emoji to assist in the later stages of semantic processing, as one might imagine readers doing if an exclamation mark was placed only at the start of a sentence rather than at the end (its conventional place in English). This would indicate that the novel position of the emoji disrupted the reader’s semantic integration during sentence wrap-up. Placing emoji in a sentence-final position is much more common and the longer fixations could be the result of sentence wrap-up effects, suggesting that readers needed to allocate additional attention to integrate the meaning of the emoji with the meaning of the message they had just read.

Taken together, these experiments suggest that the reader invests time to integrate the emoji into the text. This, in turn, might indicate that the emotional valence of an emoji could impact, and even potentially change, the meaning of a text message. Contrary to this, Robus et al. (2020) did not find significant effects related to emoji valence. This may be due to their use of narrative-like sentences rather than text messages. If the reader views the use of emoji as appropriate only within the context of CMC, then pairing emoji with sentences not presented as CMC may fail to activate their emotional valence. Support for this claim comes from earlier work on punctuation in texting, which demonstrated that periods are effective cues for conveying insincerity in text messages, but not in hand-written notes (Gunraj et al., 2016).

Additional research suggests that emoticons can influence emotional interpretation, although there are discrepancies. For example, in the study of Lo (2008), participants saw three types of messages. For one type, messages were not paired with emoticons. For the other two types, messages were paired with one of two opposite emoticons [e.g.,:-) or:-(]. Participants rated the perceived emotion of the message on a nine-point scale. Lo found that including an emoticon within a message affected both the perception and the interpretation of the message. Walther and D’Addario (2001) also found that emoticons affect the interpretation of messages, but only when negative messages were paired with negative emoticons.

More recently, Hand et al. (2022) expanded on work by Boutet et al. (2021) and found that both face emoji and object emoji can influence message emotionality and clarity, as well as the perceived warmth and emotional state of the sender. Specifically, they found that sentences paired with neutral face emoji or negative face emoji were rated more negatively than sentences paired with positive face emoji or no emoji, and that sentences paired with object emoji were rated more positively than those paired with negative face emoji, neutral face emoji, or no emoji (Hand et al., 2022). Notably, they also found that text-only messages were rated as more clear than messages combined with any type of emoji (i.e., face or object). As for sender warmth, Hand and colleagues found that the inclusion of an object emoji or a positive face emoji increased the perception of sender warmth relative to no emoji, whereas, the inclusion of a neutral face emoji or a negative face emoji decreased the perception of sender warmth relative to no emoji. Although it is clear that emoji play a role in text comprehension, it may be that when the valence of the emoticon/emoji (whether positive or negative) matches the valence of the text message, together they strengthen the emotional interpretation made by the recipient. On the other hand, it remains possible that although emoticons/emoji increase the salience of a text, they do not actually affect its interpretation.

The current study further investigates the relationship between face emoji valence and text message interpretation. We asked whether pairing texts with positively valenced emoji or negatively valenced emoji will impact the interpretation of those texts. Furthermore, we explored whether negative emoji allow for more nuanced and subtle communication of emotional information than positive emoji.

To ensure that the text messages and face emoji used as stimuli conveyed the intended tone and emotion, respectively, materials were first normed in Norming Procedure 1. Participants rated the tone of the texts on a scale of 1 (Very Negative) to 5 (Very Positive) to determine how the text messages were interpreted on their own, without the inclusion of the face emoji. Participants also rated the face emoji on two dimensions; they were asked to name the emotion conveyed by each emoji and to identify whether that emotion was mild or strong in intensity.

In Experiment 1, we used these normed materials to explore how face emoji impact the interpretation of texts. Sentences rated as neutral were paired with each of our four face emoji categories (strong positive, mild positive, strong negative, or mild negative). Participants were asked to read the text/face emoji pairs as though they had received them from someone they knew well and then to rate the tone of the text/face emoji pairs on a scale of 1 (Very Negative) to 5 (Very Positive). We expected (1) that texts paired with positive emoji would be rated more positively than texts paired with negative emoji and (2) that texts paired with mild emoji would be rated closer to neutral than texts paired with strong emoji.

In Experiment 2, we investigated whether (1) non-neutral texts can be influenced by face emoji valence and (2) negative face emoji convey more nuanced, gradated information than positive face emoji. We paired slightly positive texts with mild positive face emoji or strong positive face emoji and slightly negative texts with mild negative face emoji or strong negative face emoji. Again, participants rated the tone of the text/face emoji pairs on a scale of 1 (Very Negative) to 5 (Very Positive). We expected (1) that texts paired with positive face emoji would be rated similarly, regardless of whether the face emoji were mild or strong but (2) that texts paired with negative face emoji would be rated more negatively when the face emoji were strong compared to when the face emoji were mild.

## Norming procedure 1

In preparation for Experiment 1, we began with a norming procedure. The first portion of the norming procedure was meant to help us select texts that were neutral in tone. Participants rated the tone of individual texts that were written to be neutral on a scale of 1 (Very Negative) to 5 (Very Positive) to allow us to determine how each text would be interpreted without the inclusion of a face emoji. The second portion of the norming procedure was meant to help us select face emoji that were consistently used to express either positive emotion or negative emotion. Participants rated individual face emoji on two dimensions; they were asked to (1) name the emotion conveyed by each face emoji and (2) identify whether the intensity of that emotion was mild or strong.

The neutral texts, mild positive face emoji, strong positive face emoji, mild negative face emoji, and strong negative face emoji identified in the norming procedure were used to create the experimental stimuli in Experiment 1 (See Appendix A).

### Methods

#### Participants

One hundred ten James Madison University undergraduates voluntarily participated in this procedure in exchange for course credit. Following their participation, we asked them to complete a brief demographic questionnaire. We received 92 responses. Of the participants who answered the age question, 41 were 18 years old, 36 were 19 years old, 11 were 20 years old, three were 21 years old, and one was 26 years old (*M* = 18.82, *SD* = 1.10). Of the participants who answered the gender question, 58 (63.04%) identified as female and 34 identified as male. Of the participants who answered the item, “Would you consider yourself proficient in texting?” 78 (84.78%) said “Yes” and 14 said “No.”

#### Materials

##### Texts

Fifty-one texts were created for this task, of which 35 were written to be neutral in tone, eight to be positive, and eight to be negative. Participants were instructed to, “Imagine you received this text from someone you know well. Think about how it would make you feel. How would you rate the tone of the message?” Then, they were asked to rate each text on a scale of 1 (Very Negative) to 5 (Very Positive).

##### Emoji

Thirty face emoji, styled to match those used on Apple devices, were selected for this task [e.g., (Beaming Face with Smiling Eyes), (Angry Face)]. Due to copyright restrictions, text descriptions of the emoji are included throughout this manuscript, and not the images (Emojipedia, 2023). Participants were asked to name in one word the emotion conveyed by each face emoji (e.g., happy, mad, and sad) and to identify whether that emotion was mild or strong. This was done to ensure that emoji were appropriately classified into one of four categories for use in future experiments: strong positive emoji, strong negative emoji, mild positive emoji, and mild negative emoji.

#### Procedure

QuestionPro online survey software (Question Pro, 2022) was used to present the materials for norming. Participants first reviewed the experiment instructions and submitted their informed consent. Then, they rated the texts. All texts were shown to all participants and presented in random order. Finally, they rated the face emoji. All face emoji were shown to all participants and presented in random order.

### Results

One face emoji was eliminated due to a loading error. One participant was eliminated for failing to follow instructions. Any participant who did not rate 10% or more of the items in a section (text message or face emoji) was eliminated entirely from that section. For the text message section, 14 of 108 participants were removed, leaving 94 participants; for the face emoji section, 21 of the 108 participants were removed, leaving 87 participants.

We calculated the average rating for each text. A text was discarded if the majority of its ratings was not “3 (Neutral)” or if the average of its ratings was too far from “3 (Neutral),” which we defined as outside the range of 2.6–3.4. Twenty-five texts were retained, of which 24 were selected for use in Experiment 1.

We also calculated the average rating for each face emoji. The two face emoji that had the highest averages between 3 and 5 and were generally named with a positive word became our strong positive face emoji for Experiment 1. The two face emoji that had the lowest averages between 3 and 5 and were generally named with a positive word became our mild positive face emoji. The two face emoji that had the lowest averages between 1 and 3 and were generally named with a negative word became our strong negative face emoji. Finally, the two face emoji that had the highest averages between 1 and 3 and were generally named with a negative word became our mild negative face emoji.

In addition to the valence, the arousal inherent in each emoji is an important consideration as well. Valence values, taken from Ferré et al. (2022), appear to be consistent across our positive face emoji as well as our negative face emoji, whereas arousal values tend to be somewhat higher for the strong face emoji relative to their mild face emoji counterparts: For the strong positive face emoji (Rolling on the Floor Laughing), the mean valence was 7.96 (*SD* = 1.19) and the mean arousal was 6.68 (*SD* = 2.28). For the strong positive face emoji (Beaming Face with Smiling Eyes), the mean valence was 7.73 (*SD* = 1.14) and the mean arousal was 5.48 (*SD* = 2.36). For the mild positive face emoji (Face Savoring Food), the mean valence was 7.59 (*SD* = 1.15) and the mean arousal was 4.53 (*SD* = 2.52). For the mild positive face emoji (Grinning Face with Big Eyes), the mean valence was 7.77 (*SD* = 1.19) and the mean arousal was 4.52 (*SD* = 2.53). For the strong negative face emoji (Face with Symbols on Mouth), the mean valence was 2.82 (*SD* = 1.86) and the mean arousal was 6.71 (*SD* = 1.92). For the strong negative face emoji (Angry Face), the mean valence was 3.32 (*SD* = 1.85) and the mean arousal was 6.07 (*SD* = 2.04). For the mild negative face emoji (Confused Face), the mean valence was 2.63 (*SD* = 1.35) and the mean arousal was (*SD* 5.07 = 1.89). For the mild negative face emoji (Slightly Frowning Face), the mean valence was 2.60 (*SD* = 1.25) and the mean arousal was (*SD* 5.90 = 2.08).

## Experiment 1

In Experiment 1, we used materials from Norming Procedure 1 to explore how texters use face emoji valence to inform text comprehension. Sentences rated as neutral were paired with one of each category of face emoji (strong positive, mild positive, strong negative, and mild negative). Participants were asked to read the text/face emoji pairs as though they had received them from someone they knew well and to rate the tone of the text/face emoji pairs on a scale of 1 (Very Negative) to 5 (Very Positive), similar to what was done in the norming procedure.

We expected that texts paired with positive face emoji would be rated more positively than texts paired with negative face emoji. In other words, ratings for texts paired with positive face emoji would cluster around 4 and 5 on the scale, whereas ratings for texts paired with negative face emoji would cluster around 1 and 2. We also expected that texts paired with strong face emoji would be rated more strongly than texts paired with mild face emoji. In other words, ratings for texts paired with strong face emoji would cluster around 1 and 5, whereas ratings for texts paired with mild face emoji would cluster around 2 and 4.

### Methods

#### Participants

One hundred James Madison University undergraduates voluntarily participated in this experiment in exchange for course credit. Due to experimenter error, the demographic questionnaire was unintentionally excluded.

#### Materials

We selected 24 texts and two strong positive face emoji [(Rolling on the Floor Laughing), (Beaming Face with Smiling Eyes)], two strong negative face emoji [(Face with Symbols on Mouth), (Angry Face)], two mild positive face emoji [(Face Savoring Food), (Grinning Face with Big Eyes)], and two mild negative face emoji [(Confused Face), (Slightly Frowning Face)], from Norming Procedure 1 to create our text/face emoji pairs (See Appendix A). For each of the four face emoji categories (strong positive, strong negative, mild positive, and mild negative), 12 of the 24 texts were paired with one of the two possible face emoji from the category [e.g., (Rolling on the Floor Laughing) for strong positive], while the remaining 12 texts were paired with the remaining face emoji from that same category [e.g., (Beaming Face with Smiling Eyes) for strong positive]. This resulted in 24 texts with strong positive face emoji, 24 texts with strong negative face emoji, 24 texts with mild positive face emoji, and 24 texts with mild negative face emoji, for a total of 96 text/face emoji pairs.

In addition, we created 16 filler text/face emoji pairs, eight of which were positively valenced texts paired with our strong positive and mild positive face emoji [(Rolling on the Floor Laughing), (Beaming Face with Smiling Eyes), (Face Savoring Food), (Grinning Face with Big Eyes), with each emoji shown twice] and eight of which were negatively valenced texts paired with our strong negative and mild negative face emoji [(Face with Symbols on Mouth), (Angry Face), (Confused Face), (Slightly Frowning Face), with each emoji shown twice]. The text/face emoji pairs were designed with an online text message generator (iMessage Fake Chat, 2023) to look like screenshots of smartphones.

#### Design

Had we presented all stimuli to all participants, each participant would have seen each text four times, paired with a different face emoji each time. This could have alerted them to the manipulation, skewing our results. To minimize this likelihood, we decided to present a subset of the stimuli to each participant. Unfortunately, our software did not allow for this option. Thus, we created four stimuli sets to ensure that: (1) each experimental pair was seen by one-fourth of participants, (2) each participant saw one-fourth of experimental pairs, (3) each participant saw each text selected from Norming Procedure 1 only one time, and (4) each participant saw each face emoji selected from Norming Procedure 1 three times. Each stimuli set included 24 of the experimental pairs, all eight positive fillers, and all eight negative fillers. Participants were randomly assigned to a stimuli set, and within a stimuli set, text/face emoji pairs were randomly presented.

#### Procedure

After reviewing the experiment instructions and submitting their informed consent, participants were asked to read the text/face emoji pairs as though they had received them from someone they knew well and to rate the tone of the text/face emoji pairs on a scale of 1 (Very Negative) to 5 (Very Positive). Unlike Norming Procedure 1, where the texts were rated separately from the emoji, here, the texts and the emoji were paired together and thus, rated together. As with Norming Procedure 1, Experiment 1 utilized QuestionPro online survey software (Question Pro, 2022).

### Results and discussion

An alpha level of 0.05 was used for all analyses. All analyses were conducted with participants as a random-effect variable (*t₁*) and items as a random-effect variable (*t₂*). No data were discarded for this experiment.

As anticipated, texts paired with a positive face emoji (*M₁* = 3.78, *SD₁* = 0.41) were rated higher than texts paired with a negative face emoji (*M₁* = 2.15, *SD₁* = 0.36): *t₁*(99) = 26.77, *SEM₁* = 0.06, *p₁* < 0.001, Cohen’s *d_1_* = 2.68; *t₂*(23) = 23.64, *SEM₂* = 0.07, *p₂* < 0.001, Cohen’s *d_2_* = 4.83. Thus, when participants saw a neutral text followed by a positive face emoji, they were more likely to rate the message as positive than when they saw that same neutral text followed by a negative face emoji.

Additionally, texts paired with mild positive face emoji (*M₁* = 3.87, *SD₁* = 0.47) were rated higher than texts paired with strong positive face emoji (*M₁* = 3.70, *SD₁* = 0.47): *t₁*(99) = 3.87, *SEM₁* = 0.04, *p₁* < 0.001, Cohen’s *d₁* = 0.39; *t_2_*(23) = 2.16, *SEM₂* = 0.08, *p₂* < 0.05, Cohen’s *d_2_* = 0.44. Counterintuitively, this indicates that texts paired with strong positive face emoji were rated more negatively than texts paired with mild positive face emoji. Although the underlying cause for this is not clear, we suspect that there are instances when too much positivity might be interpreted as sarcasm, and therefore, judged to be negative rather than positive. Texts paired with mild negative face emoji (*M₁* = 2.39, *SD₁* = 0.40) were rated higher than texts paired with strong negative face emoji (*M₁* = 1.92, *SD₁* = 0.44): *t₁*(99) = 10.71, *SEM₁* = 0.04, *p₁* < 0.001, Cohen’s *d₁* = 1.07; *t_2_*(23) = 7.93, *SEM_2_* = 0.06, *p_2_* < 0.001, Cohen’s *d_2_* = 1.62. This indicates that texts paired with strong negative face emoji were rated more negatively than texts paired with mild negative face emoji.

**Figure 1:**
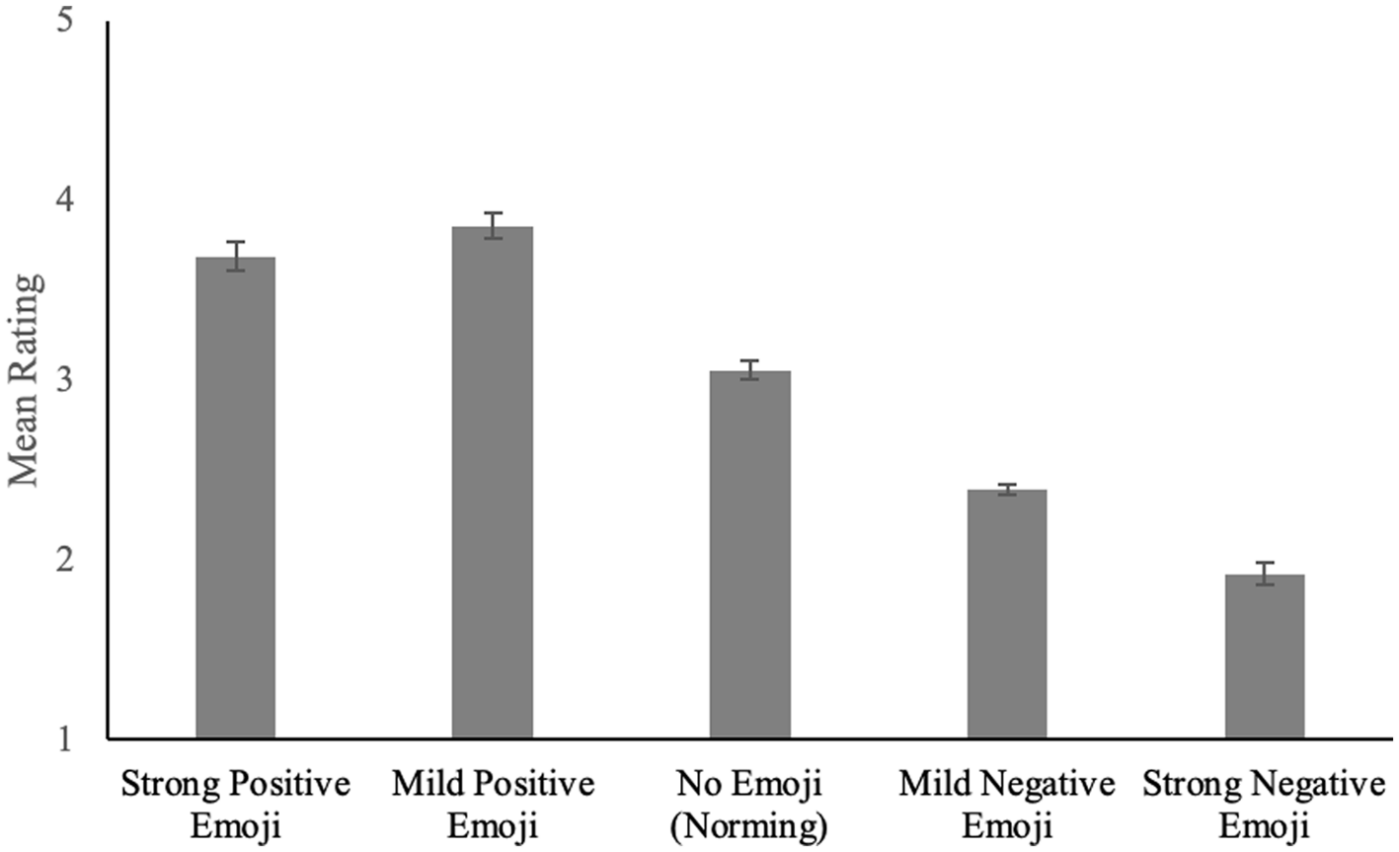
Rating of neutral texts as a function of emoji category. Error bars represent standard error (SE).

To better understand how the valence of the face emoji impacted the interpretation of the text messages, we compared the text message ratings from Norming Procedure 1 to those from Experiment 1. Recall that in the norming procedure, the text messages were presented in isolation—that is, they were not paired with the face emoji. For Norming Procedure 1/Experiment 1, the texts paired with no emoji were rated significantly different from the texts paired with emoji (see Figure 1). Texts paired with mild positive face emoji (*M_1_* = 3.87, *SD_1_* = 0.47) were rated more positively than those paired with strong positive face emoji (*M_1_* = 3.70, *SD_1_* = 0.47), which were rated more positively than those paired with no emoji (i.e., those from Norming Procedure 1; *M_1_* = 3.06, *SD_1_* = 0.21), which were rated more positively than those paired with mild negative face emoji (*M_1_* = 2.39, *SD_1_* = 0.40), which, in turn, were rated more positively than those paired with strong negative face emoji (*M_1_* = 1.92, *SD_1_* = 0.44). Thus, when paired with positive face emoji, neutral texts from the norming saw a statistically significant increase in their ratings [i.e., they were rated more positively; Strong Positive vs. No Emoji: *t_1_*(192) = 12.12, *SEM_1_* = 0.05, *p_1_* < 0.001, Cohen’s *d_1_* = 1.76; *t_2_*(23) = 9.04, *SEM_2_* = 0.07, *p_2_* < 0.001, Cohen’s *d_2_* = 2.05; Mild Positive vs. No Emoji: *t_1_*(192) = 15.46, *SEM_1_* = 0.05, *p_1_* < 0.001, Cohen’s *d_1_* = 2.23; *t_2_*(23) = 14.72, *SEM_2_* = 0.05, *p_2_* < 0.001, Cohen’s *d_2_* = 2.70] and when paired with negative face emoji, those same neutral texts saw a statistically significant decrease in their ratings [i.e., they were rated more negatively; Strong Negative vs. No Emoji: *t_1_*(192) = 22.82, *SEM_1_* = 0.05, *p_1_* < 0.001, Cohen’s *d_1_* = 3.31; *t_2_*(23) = 17.49, *SEM_2_* = 0.06, *p_2_* < 0.001, Cohen’s *d_2_* = 4.18; Mild Negative vs. No Emoji: *t_1_*(192) = 14.51, *SEM_1_* = 0.05, *p_1_* < 0.001, Cohen’s *d_1_* = 2.10; *t_2_*(23) = 12.51, *SEM_2_* = 0.05, *p_2_* < 0.001, Cohen’s *d_2_* = 3.52].

Before we conducted our next series of analyses, we converted participants’ Experiment 1 responses from a 1–5 scale to a 0–2 scale. This allowed us to determine whether texts paired with strong face emoji were rated as less neutral than texts paired with mild face emoji, independent of valence. Ratings of “3″ were converted to “0,” ratings of “2” and “4” were converted to “1,” and ratings of “1” and “5” were converted to “2.” In other words, neutral ratings (3) were set to “0,” less extreme ratings were set to “1,” and more extreme ratings were set to “2.”

As predicted, texts paired with a strong face emoji (*M₁* = 0.99, *SD₁* = 0.33) were rated as less neutral than texts paired with a mild face emoji (*M₁* = 0.81, *SD₁* = 0.28): *t₁*(99) = 6.15, *SEM_1_* = 0.03, *p₁* < 0.001, Cohen’s *d₁* = 0.62; *t₂*(23) = 3.79, *SEM_2_* = 0.04, *p₂* < 0.001, Cohen’s *d_2_* = 0.77. Thus, when participants saw a neutral text followed by a strong face emoji, they were more likely to rate the message as having a stronger valence (whether positive or negative) than when they saw that same neutral text followed by a mild face emoji.

Furthermore, texts paired with mild positive face emoji (*M₁* = 0.91, *SD₁* = 0.45) were rated higher than texts paired with mild negative face emoji (*M₁* = 0.71, *SD₁* = 0.32): *t₁*(99) = 3.63, *SEM₁* = 0.05, *p₁* < 0.001, Cohen’s *d₁* = 0.36; *t_2_*(23) = 3.26, *SEM_2_* = 0.06, *p₂* < 0.01, Cohen’s *d_2_* = 0.67. This indicates that texts paired with mild positive face emoji were rated as less neutral than texts paired with mild negative face emoji. Texts paired with strong negative face emoji (*M₁* = 1.14, *SD₁* = 0.40) were rated higher than texts paired with strong positive face emoji (*M₁* = 0.84, *SD₁* = 0.41): *t₁*(99) = 6.26, *SEM₁* = 0.05, *p₁* < 0.001, Cohen’s *d₁* = 0.63; *t_2_*(23) = 3.71, *SEM_2_* = 0.08, *p_2_* < 0.01, Cohen’s *d_2_* = 0.76. This indicates that texts paired with strong negative face emoji were rated as less neutral than texts paired with strong positive face emoji. Thus, texts paired with positive face emoji (whether mild or strong) tended to cluster closer together than texts paired with negative face emoji.

Taken together, the results indicate that the emotional valence of a face emoji can impact the interpretation of a text. The same texts that were rated as neutral in our norming procedure were rated as positive when paired with a positive emoji and negative when paired with a negative emoji. Furthermore, those same messages were rated closer to neutral when paired with a mild emoji and further from neutral when paired with a strong emoji. Work by Boutet et al. (2021) suggests that neutral texts, without an obvious emotional overtone, are especially malleable to the influence of an emoji’s emotional valence; they found the presence of an emotionally-valenced face emoji influenced participants’ perceptions of a message. Similarly, they found that the perceived warmth of the sender was positively influenced by the inclusion of a positive face emoji when paired with a neutral text message. These results support the claim that texters have found ways to adapt to their communicative environment, using face emoji to convey intention and meaning that would otherwise be expressed by face-to-face cues in spoken conversation. However, most texts are not entirely neutral and are likely to carry some valence information. Given the findings of Experiment 1, and the findings of both Boutet et al. (2021) and Hand et al. (2022) which indicate that the emotional valence of emoji influence the perceived warmth and emotional state of its sender, we next investigated the potential for face emoji to influence the interpretation of non-neutral text messages. In Experiment 2, we explore the effects of face emoji valence on slightly positive text messages and slightly negative text messages.

## Norming procedure 2

In Experiment 1, we explored how valenced face emoji paired with neutral text messages impact recipients’ interpretation. For neutral texts, emoji appear to play a disambiguating role, helping recipients to better understand the intentions of senders. For valenced texts, emoji may play a different role; rather than disambiguating the meaning of a text, an emoji may strengthen or weaken the emotional valence already conveyed by that text. Given the results of Experiment 1, where the difference in effect size was larger for the negative stimuli than for the positive stimuli, we expect that this potential modulating function may be more pronounced for negatively valenced texts than for positively valenced texts. Certainly, one could make the argument that interpreting subtle nuance is more important when a text seems negative than when it seems positive, as people are far more motivated to mitigate or altogether avoid a negative impact (Baumeister et al., 2001). It is also far more difficult to change a negative impression of oneself to a positive one than it is to change a positive impression to a negative one (Baumeister et al., 2001). Thus, there is reason to speculate that negatively valenced text/face emoji pairs will elicit a greater deal of attention and thought than positively valenced text/face emoji pairs, possibly resulting in different patterns for the ratings.

To investigate whether a face emoji has the potential to impact how the emotion conveyed by a slightly valenced text is interpreted, we began by conducting a second norming study. Texts were written to be interpreted as slightly positive or slightly negative. As with Norming Procedure 1 and Experiment 1, these texts were rated on a scale from 1 (Very Negative) to 5 (Very Positive). The texts selected from this norming procedure were paired with the face emoji selected from the previous norming procedure to create the stimuli used in Experiment 2.

### Methods

#### Participants

One hundred James Madison University undergraduates voluntarily participated in this procedure in exchange for course credit. Following their participation, we asked them to complete a brief demographic questionnaire. We received 98 responses. Of the participants who answered the age question, 35 were 18 years old, 44 were 19 years old, 13 were 20 years old, 5 were 21 years old, and one was 22 years old (*M* = 18.91, *SD* = 0.87). Of the participants who answered the gender question, 68 (69.39%) identified as female, 29 identified as male, and one identified as non-binary. Of the participants who answered the item, “Would you consider yourself proficient in texting?” 87 (88.78%) said “Yes” and 11 said “No.”

#### Materials

One hundred forty-six texts were created for this task, of which 73 were written to be slightly positive and 73 to be slightly negative. Again, participants were instructed to, “Imagine you received this text from someone you know well. Think about how it would make you feel. How would you rate the tone of the message?” Then, they were asked to rate each text on a scale of 1 (Very Negative) to 5 (Very Positive).

#### Procedure

QuestionPro online survey software (Question Pro, 2022) was used to present the materials for norming. Participants first reviewed the experiment instructions and submitted their informed consent. Then, they rated the texts. All texts were shown to all participants and presented in random order.

### Results

The results of this norming procedure were used to determine which texts to include as stimuli in Experiment 2. Two participants were eliminated for failing to follow instructions, leaving 98 participants. Each text had at least 97 responses; therefore, none were excluded due to lack of data. We did, however, exclude texts where the response pattern was highly variable. For the slightly negative texts, only those with an average rating between 2.1 and 2.6 in which 70% of responses fell within the “2-Slightly Negative” and “3-Neutral” range were maintained. Likewise, for the slightly positive texts, only those with an average rating between 3.4 and 3.9 in which 70% of responses fell within the “3-Neutral” and “4-Slightly Positive” range were maintained (Recall that in Norming Procedure 1, those texts whose average fell between 2.6 and 3.4 were classified as Neutral). All other texts were discarded. In total, 45 negative texts and 14 positive texts fit these criteria.

## Experiment 2

Experiment 1 demonstrated that the tone of neutral text messages is malleable and can be influenced by the emotional valence of face emoji. This is consistent with prior work by Boutet et al. (2021), which found that a participant’s perception of a sender’s warmth for valenced texts can be influenced by emoji. This effect appears to be strengthened when the valence of the text and emoji are congruent (i.e., a positive text paired with a positive emoji or a negative text paired with a negative emoji; see also Harris and Paradice, 2007; Huang et al., 2008; Lo, 2008; Luor et al., 2010; Filik et al., 2016).

In Experiment 2, we explored a similar but expanded question: If the recipient’s interpretation of a text’s valence is strengthened by the presence of a congruent emoji, can that be modulated by the intensity of the emoji? In other words, if a negative text is paired with a strong negative face emoji, will it be interpreted more negatively than a negative text paired with a mild negative face emoji? We investigated this question using the face emoji selected in Norming Procedure 1 and the valenced texts selected in Norming Procedure 2. For the slightly negative texts, we expected that those paired with strong negative face emoji would be rated more negatively than the same texts paired with mild negative face emoji. For the slightly positive texts, we expected that those paired with strong positive face emoji would be rated similarly to the same texts paired with mild positive face emoji–that is, we expected a diminished effect or no effect at all. It is possible that the difference between a mildly positive text/face emoji pair and strongly positive text/face emoji pair may not be pragmatically different for texters. Both responses are “good-enough” (e.g., Ferreira and Patson, 2007), and there is less social cost associated with not attending to the difference between a mildly positive face emoji and a strongly positive face emoji compared to a mildly negative face emoji and a strongly negative face emoji (Baumeister et al., 2001).

Rendle-Short (2015) discussed this diminished social cost in the context of adding an apology or a well-wish after a negative response to a text message. For example, if a friend invites one to a party but one has to decline to finish a paper for class, one might send the message, “Sorry, I can’t,” followed by “I hope you have a great time” with a positive emoji to soften the blow of the “no” response. Positive emoji–regardless of whether they are mild or strong–may help diminish social costs by softening a negative message, even if the meaning of the message is not positive overall. There might be motivation, however, for texters to consider the difference between a mild negative emoji and a strong negative emoji in order to mitigate a potential falling-out with a texting partner.

### Methods

#### Participants

One hundred fourteen James Madison University undergraduates voluntarily participated in this experiment in exchange for course credit. Following their participation, we asked them to complete a brief demographic questionnaire. We received 100 responses. Of the participants who answered the age question, 59 were 18 years old, 23 were 19 years old, 14 were 20 years old, and four were 21 years old (*M* = 18.63, *SD* = 0.87). Of the participants who answered the gender question, 85 (85%) identified as female and 15 identified as male. Of the participants who answered the item, “Would you consider yourself proficient in texting?” 87 (87%) said “Yes” and 13 said “No.”

#### Materials

We retained all 14 of the slightly positive texts and selected 14 of the 45 slightly negative texts from Norming Procedure 2 to be used in this experiment—for a total of 28 texts (See Appendix B). The 14 slightly negative texts were paired with one of each type of negative face emoji (strong negative, mild negative) and the 14 slightly positive texts were paired with one of each type of positive face emoji (strong positive, mild positive). These were the same face emoji used in Experiment 1. This resulted in seven texts paired with each face emoji, for a total of 56 text/face emoji pairs.

As with Experiment 1, we created 16 filler pairs, eight of which were positively valenced texts paired with positively valenced face emoji and eight of which were negatively valenced texts paired with negatively valenced face emoji. Again, the text/face emoji pairs were made to look like screenshots taken on smartphones (iMessage Fake Chat, 2023).

#### Design

We created four stimuli sets, each of which included 28 of the experimental pairs, all eight positive fillers, and all eight negative fillers. The stimuli sets were created to ensure that: (1) each experimental pair was seen by one-half of participants, (2) each participant saw one-half of experimental pairs, (3) each participant saw each text message selected from Norming Procedure 2 only once, and (4) each participant saw each face emoji used in Experiment 1 three to four times (Ideally, we would have liked to have shown each face emoji four times, but we would have needed 16 positive texts, rather than the 14 positive texts that were available to us). Participants were randomly assigned to a stimuli set, and within a stimuli set, text/face emoji pairs were randomly presented.

#### Procedure

After reviewing the experiment instructions and submitting their informed consent, participants were asked to read the text/face emoji pairs as though they had received them from someone they knew well and to rate the tone of the text/face emoji pairs on a scale of 1 (Very Negative) to 5 (Very Positive). As with the previous norming procedures and experiment, Experiment 2 used QuestionPro online survey software (Question Pro, 2022).

### Results and discussion

For the slightly negative texts, we expected that those paired with a strong negative face emoji would be rated more negatively than those paired with a mild negative face emoji. For the slightly positive texts, we expected a similar but diminished effect–or no effect of emoji strength at all.

An alpha level of 0.05 was used for all analyses. All analyses were conducted with participants as a random-effect variable (*t₁*) and items as a random-effect variable (*t₂*). No data were discarded for this experiment.

Unsurprisingly, the slightly positive texts paired with a positive face emoji (*M₁* = 3.90, *SD₁* = 0.45) were rated higher (i.e., more positively) than the slightly negative texts paired with negative face emoji (*M₁* = 1.86, *SD₁* = 0.34): *t₁*(113) = 36.69, *SEM₁* = 0.06, *p₁* < 0.001, Cohen’s *d_1_* = 3.44; *t₂*(26) = 27.30, *SEM_2_* = 0.07, *p_2_* < 0.001, Cohen’s *d_2_* = 10.32.

**Figure 2:**
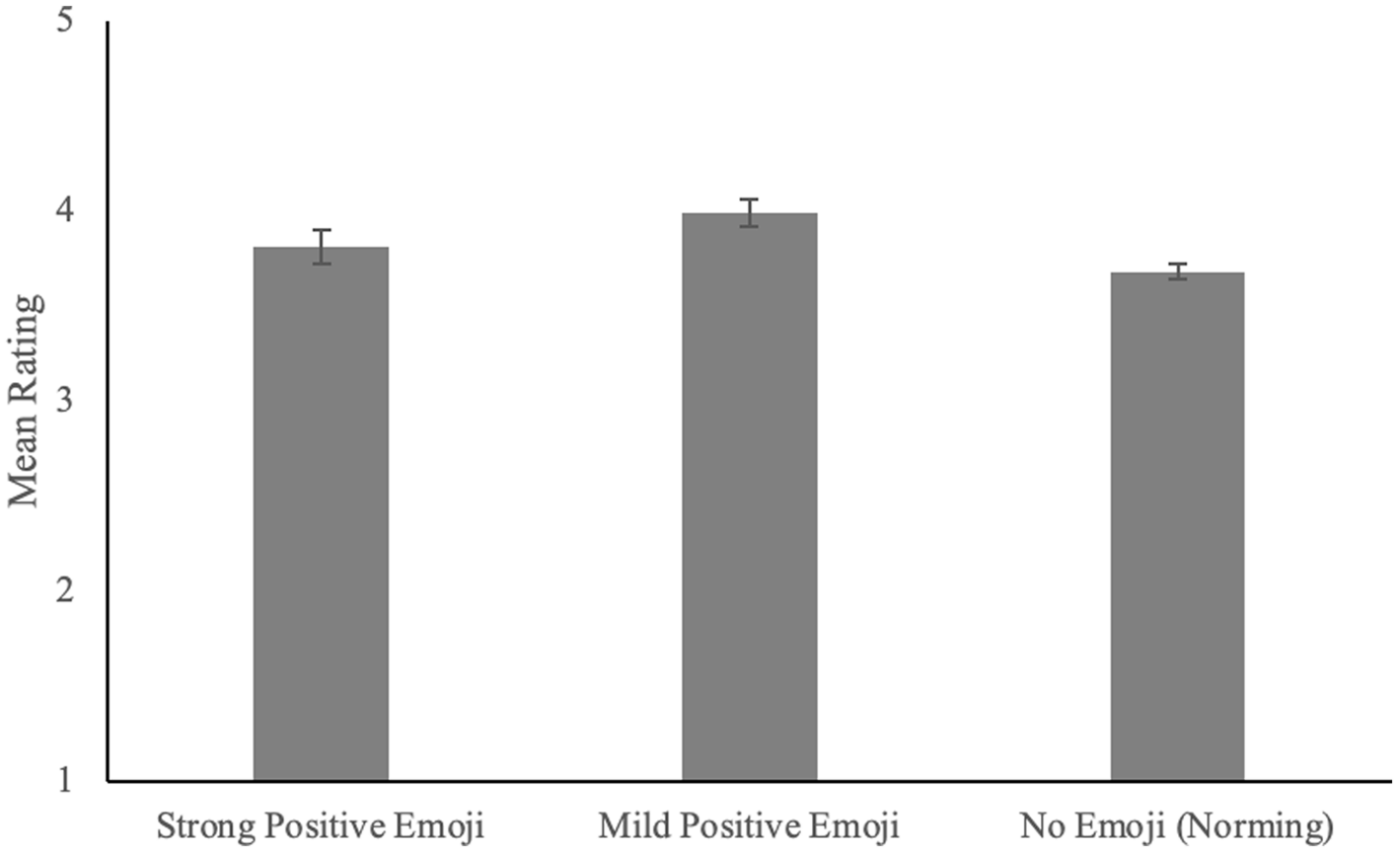
Rating of positive texts as a function of emoji category. Error bars represent standard error (SE).

Although the slightly positive texts paired with mild positive face emoji (*M₁* = 3.98, *SD₁* = 0.54) were rated higher (i.e., more positively) than the same texts paired with strong positive face emoji (*M₁* = 3.82, *SD₁* = 0.46), this difference was significant by participants only, and not by items: *t₁*(113) = 3.90, *SEM₁* = 0.04, *p₁* < 0.001, Cohen’s *d_1_* = 0.37; *t₂*(13) = 1.71, *SEM_2_* = 0.10, *p_2_* = 0.11, Cohen’s *d_2_* = 0.46. This is consistent with the explanation we put forth in Experiment 1—that texts that are seen as too positive might be interpreted as sarcastic and therefore, negative. When compared to the slightly positive texts from Norming Procedure 2 (i.e., those paired with no emoji; *M_1_* = 3.68, *SD_1_* = 0.49), those paired with mild positive face emoji were rated more positively by subjects and by items [Mild Positive vs. No Emoji: *t_1_*(210) = 4.28, *SEM_1_* = 0.07, *p_1_* < 0.001, Cohen’s *d_1_* = 0.58; *t_2_*(13) = 4.36, *SEM_2_* = 0.07, *p_2_* < 0.001, Cohen’s *d_2_* = 1.40]; those paired with strong positive face emoji were rated more positively by subjects only [Strong Positive vs. No Emoji: *t_1_*(210) = 2.12, *SEM_1_* = 0.06, *p_1_* < 0.05, Cohen’s *d_1_* = 0.29; *t_2_* (13) = 1.72, *SEM_2_* = 0.08, *p_2_* = 0.11, Cohen’s *d_2_* = 0.49; see Figure 2].

**Figure 3:**
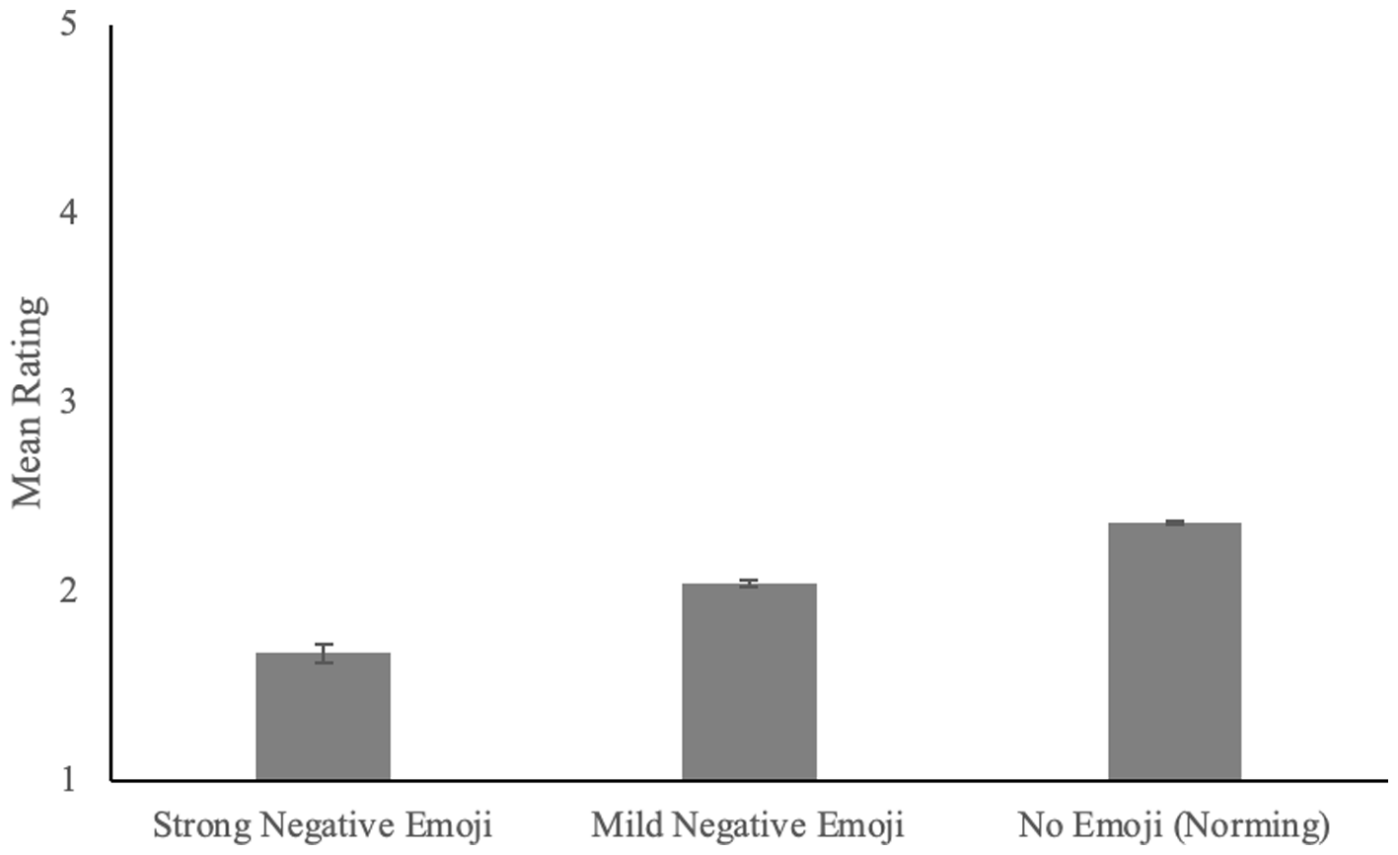
Rating of negative texts as a function of emoji category. Error bars represent standard error (SE).

For the slightly negative texts, we see a more gradated effect. Slightly negative texts paired with strong negative face emoji (*M_1_* = 1.68, *SD_1_* = 0.43) were rated more negatively than slightly negative texts paired with mild negative face emoji [*M_1_* = 2.04, *SD_1_* = 0.36; *t₁*(113) = 9.54, *SEM₁* = 0.04, *p₁* < 0.001, Cohen’s *d_1_* = 0.89; *t₂*(13) = 10.18, *SEM_2_* = 0.04, *p_2_* < 0.001, Cohen’s *d_2_* = 2.89], which were rated more negatively than slightly negative texts paired with no emoji [*M_1_* = 2.37, *SD_1_* = 0.40; Strong Negative vs. No Emoji: *t_1_*(210) = 12.15, *SEM_1_* = 0.06, *p_1_* < 0.001, Cohen’s *d_1_* = 1.66; *t_2_*(13) = 14.22, *SEM_2_* = 0.05, *p_2_* < 0.001, Cohen’s *d_2_* = 4.90; Mild Negative vs. No Emoji: *t_1_*(210) = 6.29, *SEM_1_* = 0.05, *p_1_* < 0.001, Cohen’s *d_1_* = 0.87; *t_2_*(13) = 11.38, *SEM_2_* = 0.03, *p_2_* < 0.001, Cohen’s *d_2_* = 4.18; see Figure 3].

Finally, the magnitude of the strong vs. mild effect was considerably more pronounced for the slightly negative texts (Cohen’s *d_1_* = 0.89, Cohen’s *d_2_* = 2.89) than the slightly positive texts (Cohen’s *d_1_* = 0.37, Cohen’s *d_2_* = 0.46). Thus, we have additional support suggesting that negative face emoji are more impactful and nuanced than positive face emoji when the texts they are paired with already carry valence information of their own.

## General discussion

The results of Experiments 1 and 2 provided strong evidence that both the valence and the strength of face emoji can influence the emotional interpretation of neutral text messages (Experiment 1) as well as slightly valenced text messages (Experiment 2). For both experiments, we found that our effects were more pronounced for negative face emoji than for positive face emoji. Interestingly, texts paired with mild positive face emoji were rated more positively than those same texts paired with a strong positive face emoji. Negative face emoji, on the other hand, functioned more as predicted. Texts paired with strong negative face emoji were rated more negatively than those same texts paired with mild negative face emoji. As discussed, the valence values (Ferré et al., 2022) were somewhat consistent across our positive face emoji as well as our negative face emoji. However, the arousal values tended to be slightly higher for the strong face emoji relative to the mild face emoji. Thus, the results of our experiments may demonstrate the influence of valence, arousal, and/or some combination of these two factors contributing to different degrees.

It is important to note that the difference in effect size between the strong and mild positive text/face emoji pairs was much smaller than the difference in effect size between the strong and mild negative text/face emoji pairs, indicating that although face emoji appear to carry some gradated emotional information, whether positively or negatively valenced, this was more evident for the negative face emoji. Overall, these results indicate that positive face emoji and negative face emoji are not merely two sides to the same coin, but rather, that they function differently from one another. The gradated emotional information carried by positive face emoji seems stunted compared to negative face emoji–the latter appearing communicatively richer in conveying intensity and, perhaps, meaning. This is supported by findings from Hand et al. (2022) indicating that negative emotionally congruent text/emoji pairs (i.e., pairs consisting of negative texts and negative emoji) were rated as clearer than positive emotionally congruent text/emoji pairs.

Our current findings, too, support the notion that positively valenced emoji can be somewhat difficult to interpret when paired with text messages. In isolation, the strong positive emoji were rated more positively than the mild positive emoji. However, when these emoji were paired with text messages (both neutral and slightly positively valenced), the mild pairings were rated more positively than the strong pairings. Earlier, we suggested that perhaps the strong pairings were interpreted sarcastically. We are not the first to make this observation. Some emoji, although judged as positive in isolation, show evidence of being used sarcastically, and thus, are judged as negative when paired with a text message. One common example of this involves framing a piece of bad news within the sparkles emoji or the heart emoji (Broni, 2022). Perhaps, rather than the emoji influencing the interpretation of the message, the message influences the interpretation of the emoji. Certainly, it seems that texters pay attention to the alignment between emoji valence and message content in order to intentionally subvert this relationship to communicate sarcastic or subordinate meanings (Morrissey et al., 2023; Phillips et al., 2023). Take, for example, the humorous use of the skull emoji. The skull emoji is often paired with phrases such as, “I’m dead” to figuratively indicate “dying from laughter” (e.g., Filik et al., 2016; Riordan, 2017). Exploring the ways in which texters purposefully consider and employ these greater ranges of emoji (Morrissey et al., 2023) promises to be a fruitful line of investigation.

## Conclusion

An ever-present challenge for psycholinguistics research, especially when investigating the pragmatic impact of face emoji in text messaging, involves the tradeoff between maintaining empirical precision and creating naturalistic stimuli. Despite the perhaps contrived nature of the sparse scenarios that we created for our participants to read, they were still able to immerse themselves enough to approach these artificial lab *textoids* as they would real texting conversations. Granted, our experiments presented texts involving a sender who was unknown to the participant, without providing much context for the conversation or the relationship. But the texts were presented as text bubbles on a smartphone to create a more realistic experience. This is an important distinction from earlier work where it is less clear whether participants treated the material in the experiment as text messages or other types of text/narratives (e.g., Robus et al., 2020).

Overall, the results of Experiments 1 and 2 indicate that the emotional valence of a face emoji can impact the interpretation of a text message. The same texts that were rated as neutral in our initial norming procedure were rated as positive when paired with a positive face emoji and negative when paired with a negative face emoji. Those same texts were rated closer to neutral when paired with mild face emoji and further from neutral when paired with strong face emoji. Furthermore, the results signify that negatively valenced face emoji can convey a more nuanced gradation of emotion than positively valenced face emoji. Slightly negative texts paired with strong negative face emoji were rated further from neutral than slightly positive texts paired with strong positive face emoji. Additionally, there was a greater difference in how negative texts were rated when paired with strong vs. mild face emoji compared to how positive texts were rated when paired with strong vs. mild face emoji.

Why might this difference be more pronounced for the negative face emoji? It may be that negative face emoji are subject to more scrutiny and deeper analysis by texters due to negativity dominance and the greater emotional power negative life events yield compared to positive life events (Baumeister et al., 2001; Rozin and Royzman, 2001). In other words, for texters to protect themselves and their relationships, they need to be much more accurate in how they signal and interpret negativity. Thinking that someone is *somewhat* excited about a party even though they are *very* excited is relatively harmless. Thinking that someone is *somewhat* annoyed about yet another missed dinner even though they are *very* annoyed could trigger a chain of events that leads to the end of the relationship. Thus, we conclude that positive face emoji and negative face emoji are not processed in the same way. Our work demonstrates that negative face emoji seem to convey more meaning, or are interpreted by text recipients as having more meaning, due to the nuance they assign to negative face emoji. Clearly, texters have found ways to adapt to their communicative environment, using emoji to convey intention, meaning, and subtlety that might otherwise be lost in an impoverished digital communicative environment.

## Data availability statement

The raw data supporting the conclusions of this article will be made available by the authors, without undue reservation.

## Ethics statement

The studies involving human participants were reviewed and approved by the Institutional Review Board (IRB) at James Madison University. The patients/participants provided their written informed consent to participate in this study.

## Author’s note

Portions of these data were reported at the 31st Annual Meeting of the Society for Text and Discourse and the 93rd Annual Meeting of the Eastern Psychological Association.

## Author contributions

SU: conceptualization of research, creating stimuli, creating surveys for data collection, data coding, and writing, formatting, and submission of manuscript. DG: conceptualization of research, creating stimuli, data coding, cleaning, and analysis, and writing of manuscript. NP: creating stimuli, creating surveys for data collection, data coding, and writing of manuscript. All authors contributed to the article and approved the submitted version.

## Conflict of interest

The authors declare that the research was conducted in the absence of any commercial or financial relationships that could be construed as a potential conflict of interest.

## Publisher’s note

All claims expressed in this article are solely those of the authors and do not necessarily represent those of their affiliated organizations, or those of the publisher, the editors and the reviewers. Any product that may be evaluated in this article, or claim that may be made by its manufacturer, is not guaranteed or endorsed by the publisher.

## Supplementary material

The Supplementary material for this article can be found online at: https://www.frontiersin.org/articles/10.3389/fpsyg.2023.1183299/full#supplementary-material
