# Supplementary material for: Mad or mad-mad: conveying subtle emotion with face emoji
Source: Front Psychol. 2023 Sep 28;14:1183299. doi: 10.3389/fpsyg.2023.1183299 (PMC10569492; doi:10.3389/fpsyg.2023.1183299)
Supplement: Supplementary file 1 [file Data_Sheet_1.docx]

**Appendix A**

Experiment 1 Stimuli

**Face Emoji:** selected from Norming Procedure 1, used in Experiments 1 and 2

(Note: Due to copyright restrictions text descriptions of the emoji are included and not the images.):

**Strong Positive:** [Beaming Face with Smiling Eyes] [Rolling on the Floor Laughing]

**Mild Positive:** [Grinning Face with Big Eyes] [Face Savoring Food]

**Strong Negative:** [Angry Face] [Face with Symbols on Mouth]

**Mild Negative:** [Slightly Frowning Face] [Confused Face]

**Neutral Texts:** selected from Norming Procedure 1, used in Experiment 1

1. I asked Mike to take another look at it for me
2. I just started getting dressed for the party
3. I think he can take the photos while I put together the slides
4. I think I’m going to wear the green shirt
5. I’ll probably call her tonight or tomorrow
6. I’m headed to the library
7. I didn’t hear he was back in town
8. I have a swim meet today
9. I have to do one more thing before I can head out
10. I saw her walking by earlier today
11. I saw Michelle at the gym
12. I’m headed to the store
13. I think he got that sweater as a gift
14. I think I can ride my bike there
15. I thought I saw something on his shirt
16. Kevin might ask to borrow that book
17. Nicole left her jacket in my car
18. Yea, I think so
19. I forgot what I was gonna do
20. I just remembered I put my wallet in my book bag
21. I need to get more ink for my printer
22. I took a class with him last semester
23. I’ll be there as soon as I find Chris
24. Jen said it was a 3 hour drive

**Appendix B**

Experiment 2 Stimuli

**Emoji:** selected from Norming Procedure 1, used in Experiments 1 and 2

(Note: Due to copyright restrictions text descriptions of the emoji are included and not the images.):

**Strong Positive:** [Beaming Face with Smiling Eyes] [Rolling on the Floor Laughing]

**Mild Positive:** [Grinning Face with Big Eyes] [Face Savoring Food]

**Strong Negative:** [Angry Face] [Face with Symbols on Mouth]

**Mild Negative:** [Slightly Frowning Face] [Confused Face]

**Texts:** selected from Norming Procedure 2, used in Experiment 2

**Slightly Positive Texts**

1. I have it all written down
2. I switched from soda to sparkling water
3. I’ll give you their number
4. I just started a new series on Netflix
5. I should be finished soon
6. I tried out a new recipe last night
7. We split dessert
8. My walk wasn’t far
9. The tomatoes I grew are ripe
10. There was little traffic on my commute
11. I watched the new season in one day
12. That burrito was pretty filling
13. They just called
14. That lotion is really creamy

**Slightly Negative Texts**

1. I bought the wrong size shirt
2. My clothes didn’t dry
3. The food tonight tasted weird
4. The line was much longer than usual
5. I forgot to pack shampoo
6. The power went out last night
7. There’s something stuck in my eye
8. I don’t think I can come today
9. I had a long day
10. The gym near my house is closing
11. Turns out the bowling alley will be closed on Friday night
12. I won’t be getting paid for another day or two
13. The restaurant said the wait will be an hour
14. They were out of bagels

**Appendix C**

Experiment 1 Fillers

**Strong Positive:** [Beaming Face with Smiling Eyes] [Rolling on the Floor Laughing]

**Mild Positive:** [Grinning Face with Big Eyes] [Face Savoring Food]

**Strong Negative:** [Angry Face] [Face with Symbols on Mouth]

**Mild Negative:**[Slightly Frowning Face] [Confused Face]

**Positive Fillers**

1. Good job on the presentation yesterday [Grinning Face with Big Eyes]
2. She looked so pretty in that new dress [Grinning Face with Big Eyes]
3. I’m really feeling myself today lol [Face Savoring Food]
4. Was so great to run into him last week [Face Savoring Food]
5. Hahahaha you’re hilarious [Rolling on the Floor Laughing]
6. Omg that’s so funny [Rolling on the Floor Laughing]
7. Have a great birthday! [Beaming Face with Smiling Eyes]
8. I found the perfect outfit [Beaming Face with Smiling Eyes]

**Negative Fillers**

1. I’m so sorry about that [Slightly Frowning Face]
2. My legs are so sore after going to the gym [Slightly Frowning Face]
3. I don’t think he’s good enough for you [Confused Face]
4. I forgot how unreliable she is [Confused Face]
5. I don’t even care about that anymore [Angry Face]
6. I hate how often that happens [Angry Face]
7. He’s incredibly annoying [Face with Symbols on Mouth]
8. I don’t think I’ve ever been angrier [Face with Symbols on Mouth]

**Appendix D**

Experiment 2 Fillers

**Positive Fillers**

1. I submitted my paper two days early [Face Savoring Food]
2. We got a lot of work done [Grinning Face with Big Eyes]
3. I found a new restaurant I think you’ll like [Beaming Face with Smiling Eyes]
4. I earned extra credit on the last exam [Rolling on the Floor Laughing]
5. I liked meeting your family [Grinning Face with Big Eyes]
6. Your friends were fun to talk with [Rolling on the Floor Laughing]
7. Your new dog is so cute [Face Savoring Food]
8. I can get us dinner tonight [Beaming Face with Smiling Eyes]

**Negative Fillers**

1. I can’t help you move [Confused Face]
2. I got a parking ticket yesterday [Face with Symbols on Mouth]
3. I didn’t do well on the exam [Angry Face]
4. The bus ride was annoying [Slightly Frowning Face]
5. I can't get you any more [Slightly Frowning Face]
6. There’s even more reading due this week [Angry Face]
7. Going out together sounds tiring [Confused Face]
8. My credit card charged me a late fee [Face with Symbols on Mouth]
